# piRNA-36741 regulates BMP2-mediated osteoblast differentiation via METTL3 controlled m6A modification

**DOI:** 10.18632/aging.203630

**Published:** 2021-10-13

**Authors:** Jianmin Liu, Ming Chen, Longyang Ma, Xingbo Dang, Gongliang Du

**Affiliations:** 1 Surgery Department, Shaanxi Provincial People’s Hospital, Xi’an 710068, China; 2 Department of Orthopedics, Shaanxi Provincial People’s Hospital, Xi’an 710068, China

**Keywords:** piRNA-36741, METTL3, BMP2, BMSCs, osteogenic differentiation, osteoporosis

## Abstract

The osteogenic differentiation of bone marrow mesenchymal stem cells (BMSCs) is essential for bone formation, and its imbalance can lead to bone diseases such as osteoporosis. It is reported that PIWI-interacting RNA-36741 (piR-36741) is up-regulated during the osteogenic differentiation, but its role in regulating osteogenic differentiation remains unclear. Here, the primary human BMSCs were used to induce osteogenic differentiation, and the expression of piR-36741 and METTL3 (methyltransferase like 3) was up-regulated during the osteogenic differentiation of BMSCs. Moreover, interference with piR-36741 or METTL3 markedly hindered the osteogenic differentiation of BMSCs, which was manifested by a reduction in osteoblast marker expression (including RUNX2, COL1A1, OPN and OCN), osteogenic phenotype and matrix mineralization. Mechanistically, the piR-36741-PIWIL4 complex directly interacted with METTL3 and prevented METTL3-mediated m6A modification of BMP2 mRNA transcripts, thereby promoting BMP2 expression. And overexpression of BMP2 reversed the inhibitory effect of piR-36741 silence on osteogenic differentiation and the Smad pathway activity. In addition, administration with piR-36741 mimic alleviated ovariectomy-induced osteoporosis in mice. In conclusion, piRNA-36741 overexpression promoted osteogenic differentiation of BMSCs and mitigated ovariectomy-induced osteoporosis through METTL3-mediated m6A methylation of BMP2 transcripts.

## INTRODUCTION

Osteoporosis (OP) is a systemic bone disease characterized by bone loss. It is manifested as a decrease in bone mass and structural degradation of bone tissue, resulting in impaired bone strength and increased possibility of fragility fractures [1]. The basic pathogenesis of OP is the imbalance between osteoblast bone formation and osteoclast bone resorption [2]. Bone marrow mesenchymal stem cells (BMSCs) are the precursor cells of osteoblasts, which have the ability to differentiate into multiple cell lineages (osteoblasts, chondrocytes, bone marrow stromal cells and adipocytes) and self-renewal [3]. The osteogenic differentiation ability of BMSCs can be used for bone repair and remodeling [4]. Therefore, inducing BMSCs to differentiate into bone tissue and correcting bone imbalance is one of the therapeutic directions of OP.

PIWI-interacting RNAs (piRNAs) are a type of non-coding RNA with a length of 26–31 nucleotides, which were first found in mammalian reproductive cells [5]. piRNAs can form complexes with PIWIL proteins (P-element induced wimpy testis like), members of the Argonaute family, to affect transposon silencing, genome rearrangement, spermatogenesis, protein regulation, epigenetic regulation, and reproductive stem cell maintenance [6]. Human body contains four PIWIL proteins (PIWIL1–4), and they combine about 23’439 known piRNAs [7]. Although piRNAs are generally believed to have important functions in germline development, recent evidence suggests that piRNAs are also expressed in many human tissues in a tissue-specific manner, and regulate key signaling pathways at the transcription or post-transcriptional level [8]. Growing evidence showed that piRNAs are involved in the occurrence and development of many cancers, and are also closely related to central nervous system diseases and heart regeneration [9]. In addition, a recent study showed that a variety of piRNAs are dysregulated during the osteogenic differentiation of human bone marrow mesenchymal stromal cells, in which the expression of piR-36741 is significantly up-regulated [10]. However, the specific regulatory mechanism of piR-36741 in the process of osteogenic differentiation of BMSCs remains unclear.

N6-methyladenosine (m6A) is the most common post-transcriptional internal mRNA modification that can regulate a variety of biological processes [11]. In mammals, m6A methylation is usually catalyzed by the methyltransferase METTL3 and its partners METTL14 and WTAP, and can be erased by the demethylases FTO and ALKBH5. M6A modification is mainly recognized and binding by m6A “readers”, YTHDF1–3 and YTHDC1/2 from YTH domain family, thereby promoting many biological processes, such as tumorigenesis, viral infection and adipogenic differentiation [12]. The addition of m6A affects various aspects of mRNA metabolism, such as RNA stability and translation, and splicing of pre-mRNA [13]. A recent research indicated that the loss of METTL3 function can inhibit the osteogenic differentiation potential of BMSCs, leading to impaired bone formation and bone marrow fat accumulation. And overexpression of METTL3 in BMSCs protects mice from osteoporosis caused by estrogen deficiency [14]. According to a previous report, a cardiac-hypertrophy-associated piRNA is demonstrated to inhibit the m6A modification of the target gene of METTL3 by competitively binding to METTL3 [15]. We are committed to exploring whether piR-36741 is involved in the osteogenic differentiation of BMSCs through interaction with METTL3.

In this study, we demonstrated the promoting effects of piR-36741 on osteogenic differentiation of BMSCs and the relieving effects of it on ovariectomy-induced osteoporosis. We also revealed the potential molecular mechanism of piR-36741 in regulating osteogenic differentiation, which involved METTL3-mediated m6A methylation regulation of BMP2 mRNA. These findings may contribute to the treatment of patients with postmenopausal osteoporosis.

## RESULTS

### piR-36741 and METTL3 expression was upregulated in osteogenic differentiation of BMSCs

**Figure 1:**
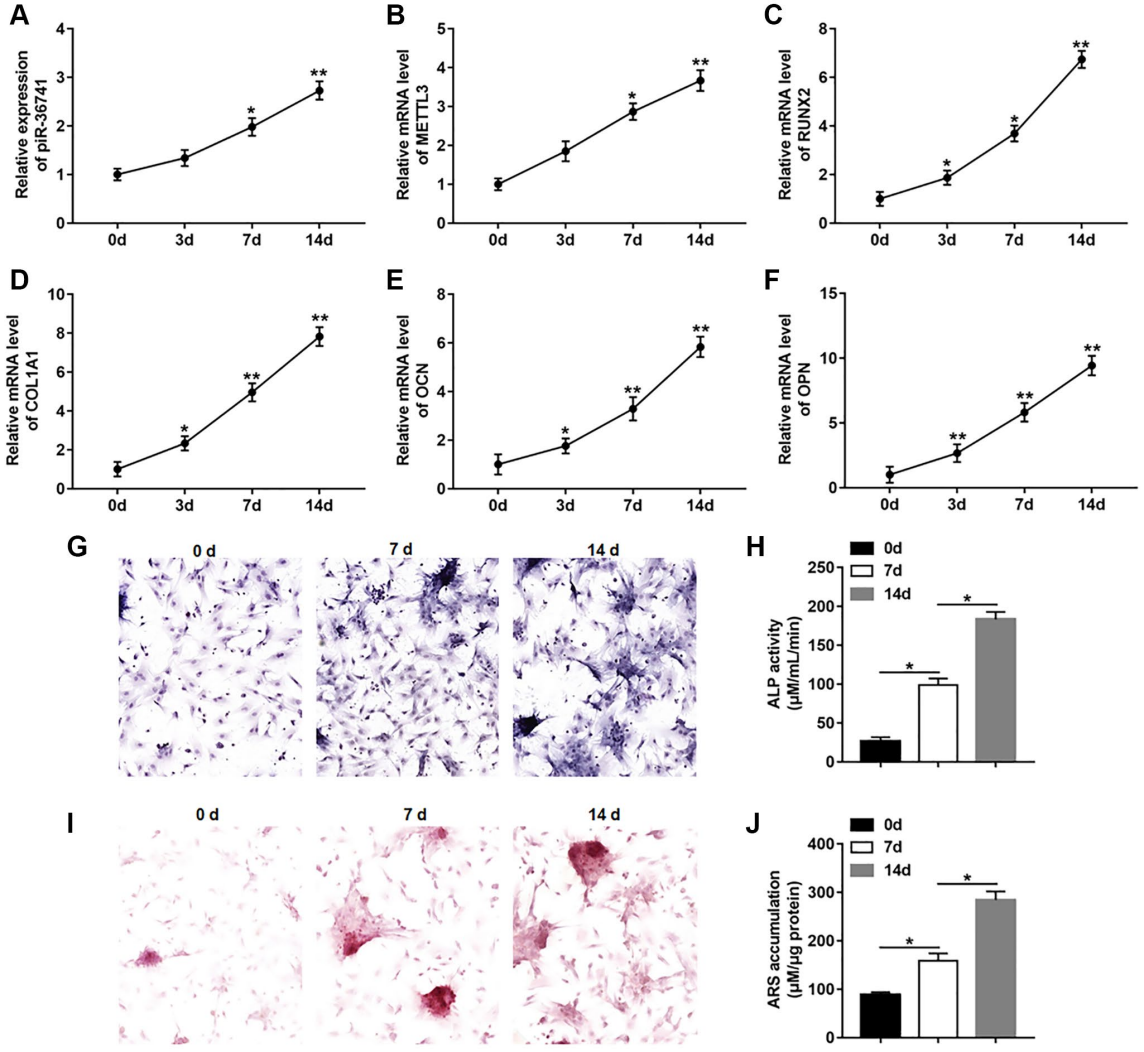
**piR-36741 and METTL3 expression was upregulated in osteogenic differentiation of BMSCs.** BMSCs were treated by 10 mM β-glycerophosphate, 100 nM dexamethasone, and 200 μM ascorbic acid to induce osteogenic differentiation. (**A**–**F**) The expression of piR-36741 (**A**), and the mRNA levels of METTL3 (**B**), RUNX2 (**C**), COL1A1 (**D**), OCN (osteocalcin) (**E**) and OPN (osteopontin) (**F**) were detected on day 0, 3, 7 and 14. (**G**) Images of ALP staining (100×). (**H**) ALP activity was measured on day 0, 7 and 14. (**I**) Images of Alizarin red S (ARS) staining (100×). (**J**) Quantitative analysis of ARS accumulation on day 0, 7 and 14. *N* = 5 in each group. ^*^P < 0.05, ^**^P < 0.01. Each test was independently repeated at least three times.

To investigate the changes of piR-36741 and METTL3 expression in osteogenic differentiation, BMSCs were treated with osteogenic induction medium for 14 days to induce osteogenic differentiation. The results displayed that the expression of piR-36741 and METTL3 was increased in the process of osteogenic differentiation (Figure 1A and 1B). And the mRNA levels of osteoblast markers RUNX2 (runt-related transcription factor 2), COL1A1 (collagen type I), OCN (osteocalcin) and OPN (osteopontin) were gradually up-regulated during osteogenesis (Figure 1C–1F). Moreover, the osteogenic phenotype was revealed by increased ALP staining and ALP activity (Figure 1G and 1H). Meanwhile, ARS staining and quantification also indicated the enhanced mineralization during osteogenic differentiation (Figure 1I and 1J).

### Silencing piR-36741 hindered osteogenic differentiation of BMSCs

**Figure 2:**
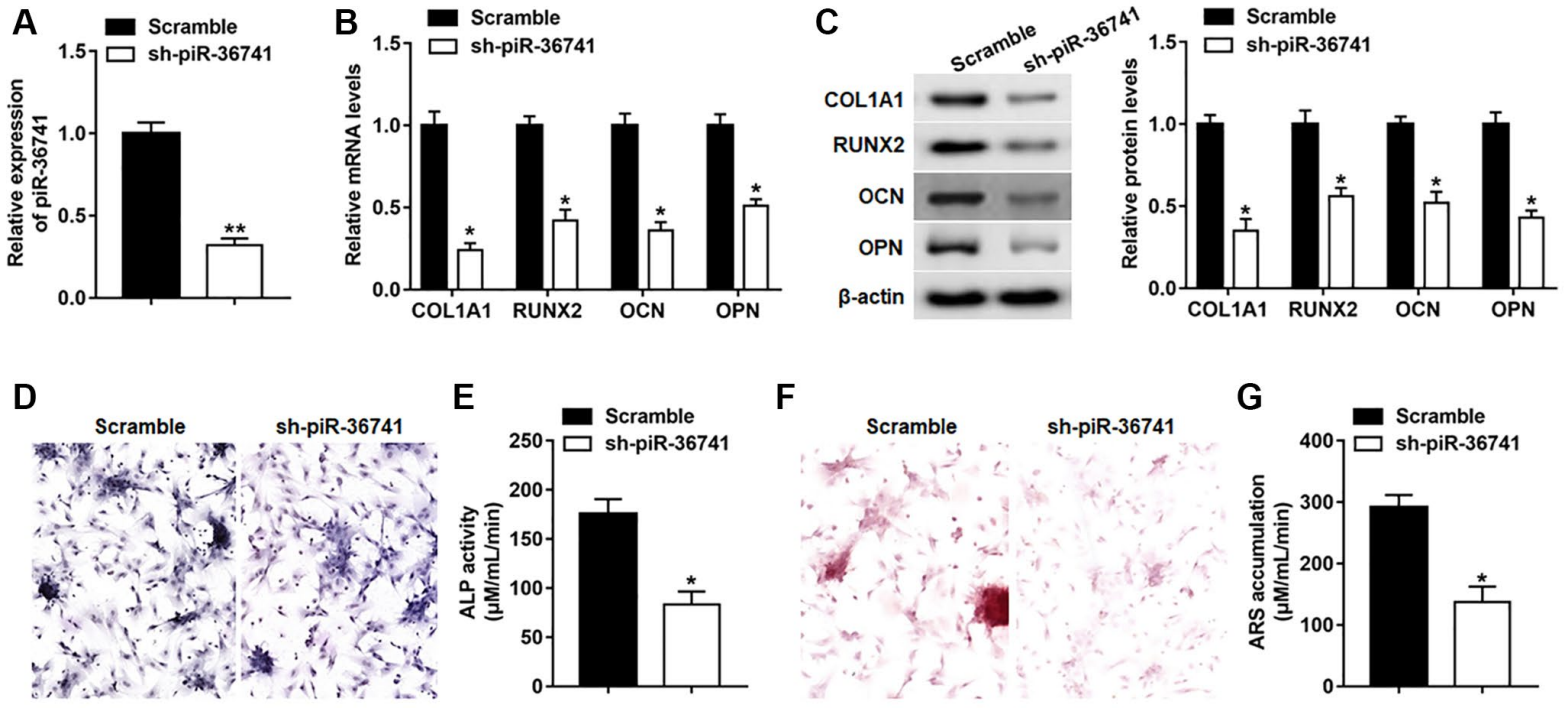
**Silencing piR-36741 hindered osteogenic differentiation of BMSCs.** BMSCs were infected with Lv-sh-NC and Lv-sh-piR-36741, and then cultured in osteogenic differentiation medium for 14 days. (**A**–**C**) The expression of piR-36741, and the mRNA and protein levels of METTL3, RUNX2, COL1A1, OCN and OPN were measured on day 14. (**D**) Images of ALP staining on day 14 (100×). (**E**) ALP activity was determined on day 14. (**F**) Images of ARS staining (100×). (**G**) Quantitative analysis of ARS accumulation on day 14. *N* = 5 in each group. ^*^P < 0.05, ^**^P < 0.01. Each test was independently repeated at least three times.

To explore the role of piR-36741 in osteogenic differentiation, BMSCs were infected with Lv-sh-NC or Lv-sh-piR-36741, and the expression of piR-36741 was significantly suppressed on day 14 (Figure 2A). The mRNA and protein expression of osteogenesis-related markers RUNX2, COL1A1, OPN and OCN was also down-regulated on day 14 after silencing piR-36741 (Figure 2B and 2C). Moreover, ALP activity and staining and ARS accumulation were significantly decreased, indicating a reduction in osteogenic phenotype and matrix mineralization (Figure 2D–2G).

### piR-36741 bound to METTL3 to regulate the m6A activity of METTL3

**Figure 3:**
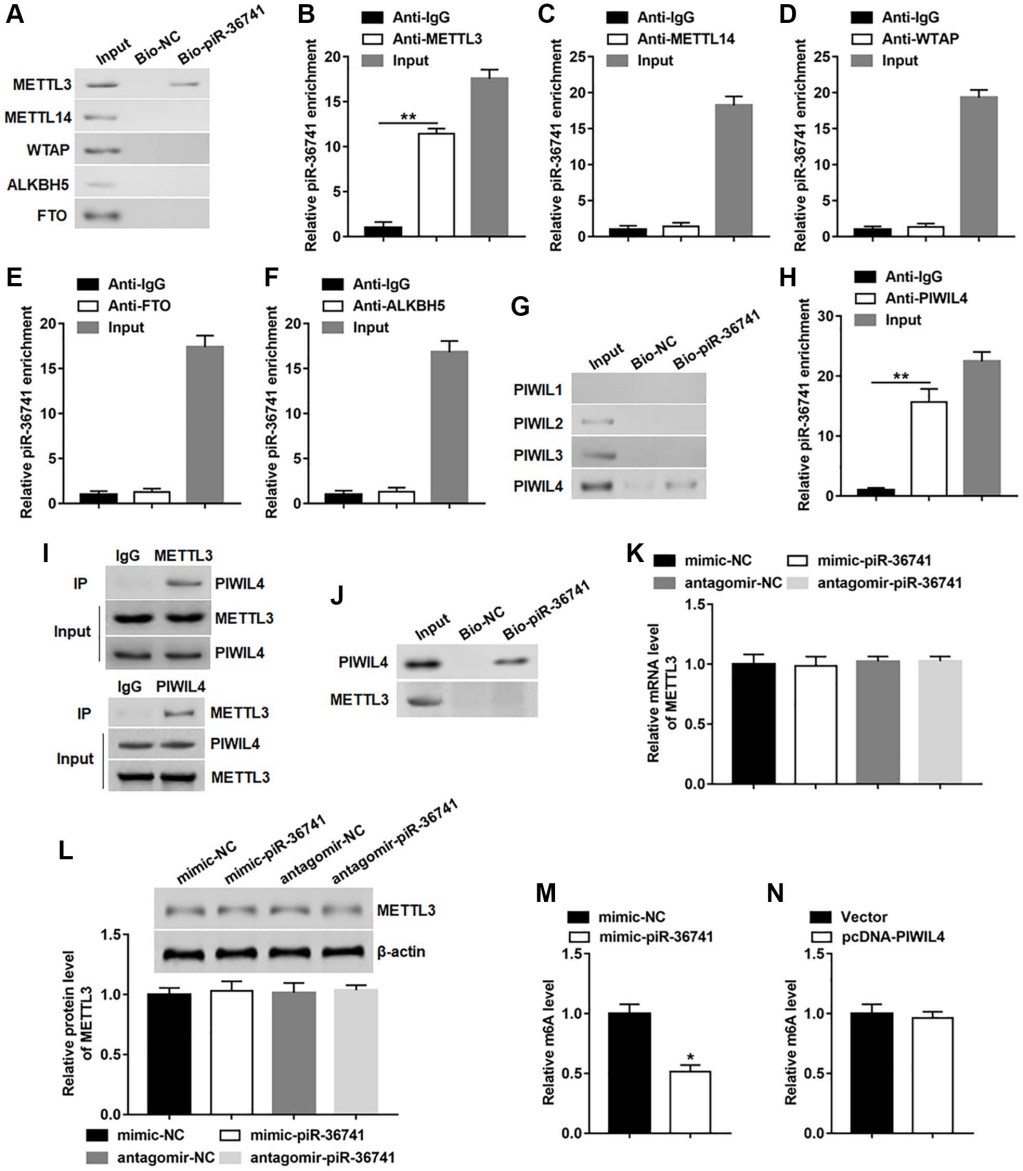
**piR-36741 bound to METTL3 to regulate the m6A activity of METTL3.** (**A**) RNA pull-down assay was performed using bio-NC or bio-piR-36741 to verify the binding of piR-36741 and METTL3. (**B**–**F**) RIP assay was carried out to confirm the binding of piR-36741 with METTL3, METTL14, WTAP, FTO and ALKBH5. (**G**) Biotin-based RNA pull-down assay was used to detect the specific binding of piR-36741 with PIWIL4. (**H**) RIP assay was performed to verify the binding piR-36741 with PIWIL4. (**I**) BMSCs were transfected with METTL3 and PIWIL4 overexpression vectors, and co-IP was carried out using specific antibodies, and the immunocomplex were purified and subjected to Western blotting analysis using METTL3 or PIWIL4 antibodies. (**J**) RNA pull-down assay was used to confirm the binding ability of purified PIWIL4 or METTL3 proteins with biotin-labelled piR-36741. (**K**, **L**) BMSCs were transfected with mimic-piR-36741, antagomir-piR-36741 or respective controls for 24 h, and the mRNA and protein levels of METTL3 were determined. (**M**, **N**) m6A levels of BMSCs with or without piR-36741/PIWIL4 overexpression were detected by using the EpiQuik™ m6A RNA methylation quantification kit. *N* = 5 in each group. ^*^P < 0.05, ^**^P < 0.01. Each test was independently repeated at least three times.

In order to further explore the mechanism of piR-36741 affecting osteogenic differentiation, RNA pull-down assay was conducted by using biotinylated piR-36741 to detect whether piR-36741 bound to the key regulatory elements of m6A. The results indicated that piR-36741 could only bind to METTL3, but not METTL14, WTAP, ALKBH5 and FTO (Figure 3A). Subsequent RIP tests confirmed that piR-36741 was enriched in METTL3, but not in other protein-RNA precipitates (Figure 3B–3F). According to a report, piRNAs can form complexes with PIWIL protein to participate in a variety of biological processes [6]. We analyzed the binding of biotinylated piR-36741 to PIWIL1–4. PiR-36741 and PIWIL-4 showed obvious binding effect, which was verified by RNA pull-down and RIP tests (Figure 3G and 3H). Besides, the co-IP results also revealed that METTL3 could interact with PIWIL-4 protein (Figure 3I). To verify whether piR-36741 could directly bind to both METTL3 and PIWIL-4, biotinylated piR-36741 and purified PIWIL-4 and METTL3 proteins were used to perform RNA pull-down experiments. The data displayed that piR-36741 bound directly to PIWIL-4 rather than METTL3 (Figure 3J). In addition, we also tested the regulatory effect of piR-36741 on METTL3 expression, and found that regardless of overexpression or interference with piR-36741, the mRNA and protein levels of METTL3 remained unchanged (Figure 3K and 3L). Notably, the global m6A level in piR-36741 overexpression group was decreased compared with that in the control group (Figure 3M). While overexpression of PIWIL4 could not alter the global m6A level (Figure 3N). These results showed that although piR-36741 could not cause changes in METTL3 expression, it could affect the m6A activity of METTL3.

### Silencing METTL3 impeded osteogenic differentiation of BMSCs

**Figure 4:**
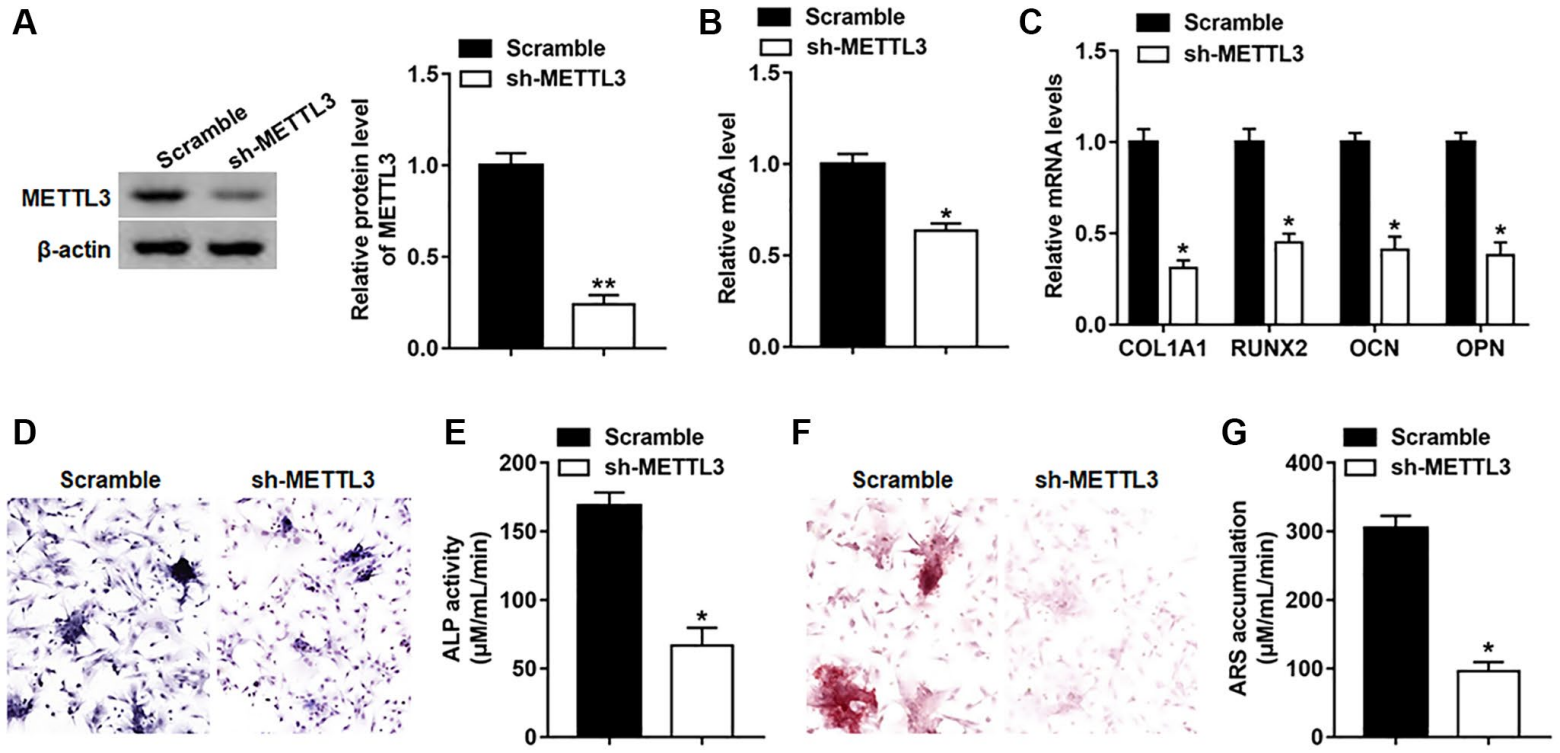
**Silencing METTL3 impeded osteogenic differentiation of BMSCs.** BMSCs were infected with Lv-sh-NC or Lv-sh-METTL3, and then cultured in osteogenic differentiation medium for 14 days. (**A**) The expression of piR-36741 was measured on day 14. (**B**) The global m6A level of BMSCs with or without METTL3 knockdown was analyzed with the EpiQuik™ m6A RNA methylation quantification kit. (**C**) The mRNA levels of METTL3, RUNX2, COL1A1, OCN and OPN were detected on day 14. (**D**) Images of ALP staining on day 14 (100×). (**E**) ALP activity was determined on day 14. (**F**) Images of ARS staining on day 14 (100×). (**G**) Quantitative analysis of ARS accumulation on day 14. *N* = 5 in each group. ^*^P < 0.05, ^**^P < 0.01. Each test was independently repeated at least three times.

To study the effect of METTL3 on osteogenic differentiation, METTL3 was suppressed by infection with lv-METTL3-shRNA, and the protein level of METTL3 was observably downregulated on day 14 (Figure 4A). Besides, silencing METTL3 decreased the global m6A level in BMSCs (Figure 4B). Moreover, the expression of RUNX2, COL1A1, OPN and OCN was declined on day 14 after suppressing METTL3 (Figure 4C). Meanwhile, osteogenic phenotype and matrix mineralization were reduced as evidenced by decreased ALP activity and staining and ARS accumulation (Figure 4D–4G).

### piR-36741 suppressed METTL3-mediated BMP2 m6A methylation to promote BMP2 expression

**Figure 5:**
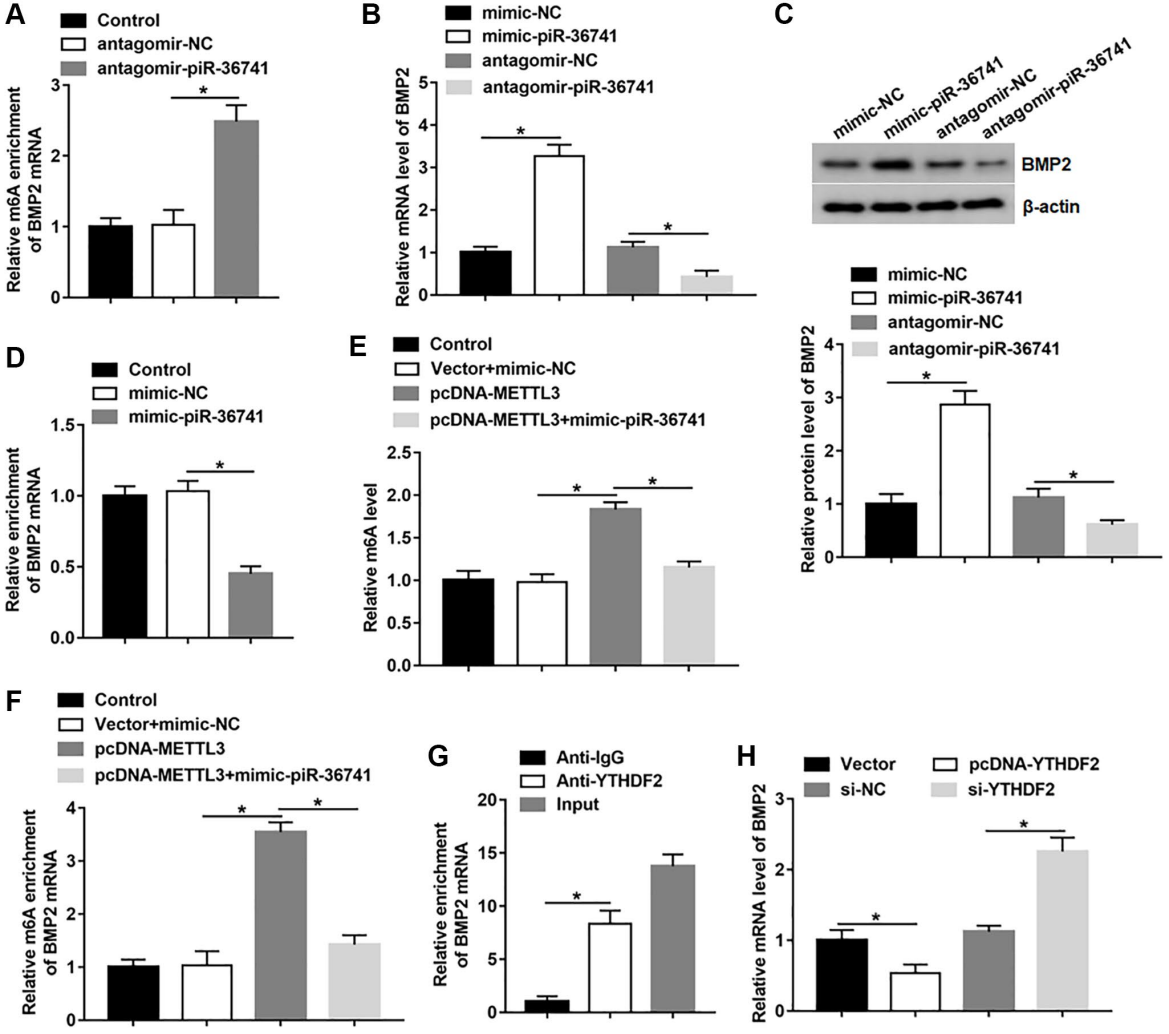
**piR-36741 suppressed METTL3-mediated BMP2 m6A methylation to promote BMP2 expression.** (**A**) The m6A level of BMP2 mRNA in BMSCs treated with antagomir-piR-36741 or antagomir-NC was detected by using MeRIP-qPCR. (**B**, **C**) The mRNA and protein levels of BMP2 in BMSCs treated with mimic-piR-36741, antagomir-piR-36741 or respective controls were measured. (**D**) RIP assay was used to analyze the level of BMP2 mRNA binding to METTL3 using anti-IgG or anti-METTL3. BMSCs were transfected with METTL3 overexpression vector alone or together with mimic-piR-36741, and (**E**) the global m6A level and (**F**) BMP2 m6A level were detected by using MeRIP-qPCR. (**G**) RIP assay was performed to confirm the binding of YTHDF2 with BMP2 mRNA. (**H**) The mRNA level of BMP2 was determined in BMSCs treated with pcDNA-YTHDF2, YTHDF2 siRNA or respective controls. *N* = 5 in each group. *P < 0.05. Each test was independently repeated at least three times.

We first studied the effect of piR-36741 on BMP2 m6A level and BMP2 expression. The results revealed that silencing piR-36741 markedly increased the m6A level of BMP2 (Figure 5A). And piR-36741 overexpression promoted the mRNA and protein levels of BMP2, and silencing piR-36741 had an opposite effect (Figure 5B and 5C). We also found that the forced expression of piR-36741 inhibited the binding of METTL3 to BMP2 mRNA (Figure 5D). And the global m6A level was upregulated after overexpressing METTL3, which was suppressed by piR-36741 overexpression (Figure 5E). Besides, overexpressing METTL3 markedly increased BMP2 m6A methylation level, which was reversed by treatment with piR-36741 mimic, suggesting that piR-36741 trapped METTL3 to prevent it from binding to BMP2 mRNA (Figure 5F). YTHDF2 is a m6A reader protein, which is mainly responsible for the degradation of m6A methylated mRNA [12]. YTHDF2 could bind to BMP2 mRNA and restrain BMP2 mRNA level (Figure 5G, 5H). Moreover, the knockdown of YTHDF2 significantly promoted BMP2 mRNA expression (Figure 5H), revealing that YTHDF2 was involved in the recognition and degradation of m6A methylated BMP2 transcripts.

### BMP2 overexpression reversed the effect of METTL3 silence on osteogenic differentiation

**Figure 6:**
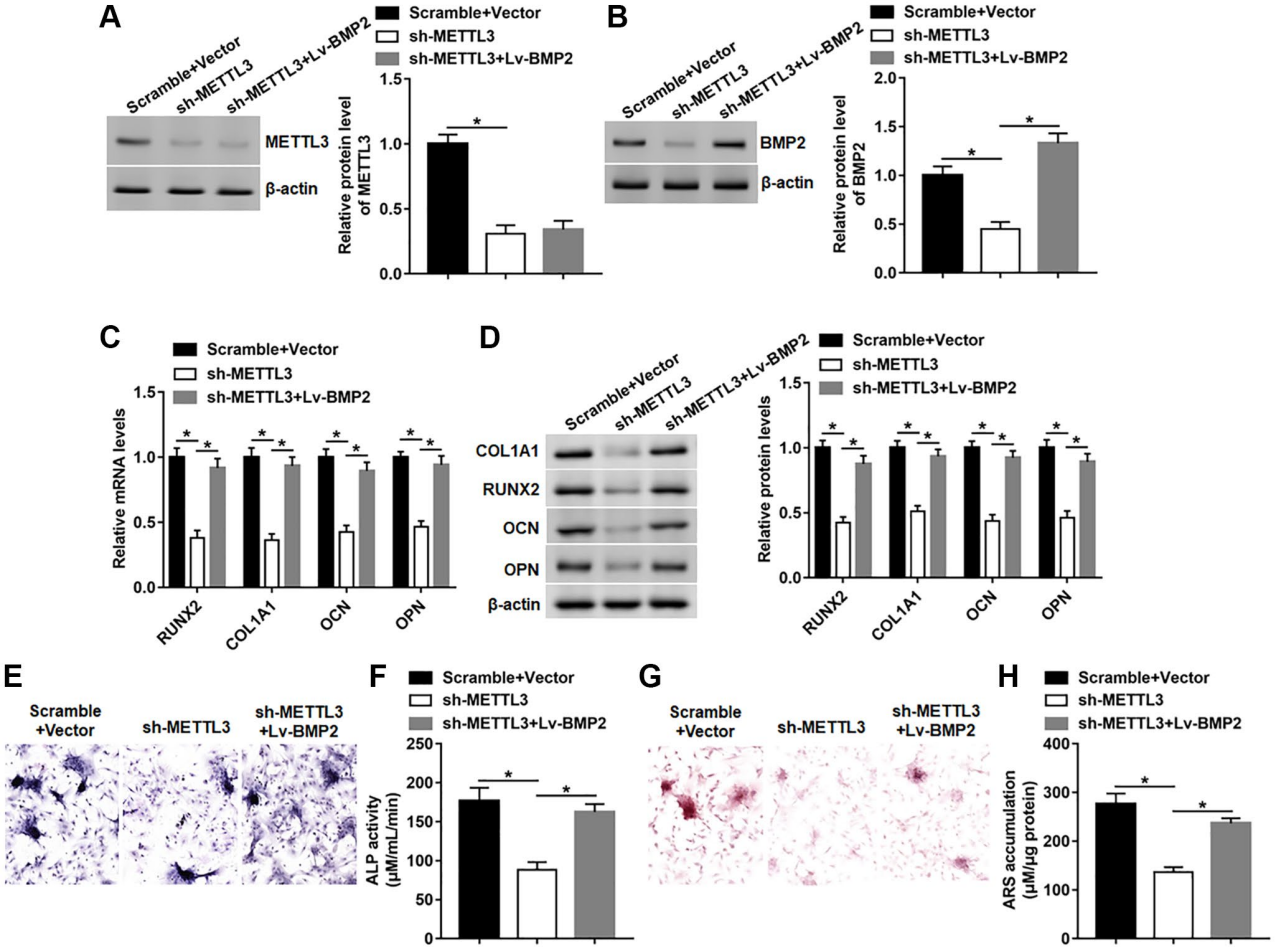
**BMP2 overexpression reversed the effect of METTL3 silence on osteogenic differentiation.** BMSCs were infected with Lv-sh-METTL3 alone or together with Lv-BMP2, and then cultured in osteogenic differentiation medium for 14 days. (**A**, **B**) The protein level of METTL3 and BMP2 were detected on day 14. (**C**, **D**) The protein expression of METTL3, RUNX2, COL1A1, OCN and OPN were assessed on day 14. (**E**) Images of ALP staining (100×). (**F**) ALP activity was measured on day 14. (**G**) Images of ARS staining (100×). (**H**) Quantitative analysis of ARS accumulation on day 14. *N* = 5 in each group. ^*^P < 0.05. Each test was independently repeated at least three times.

Next, we explored whether BMP2 overexpression affected the inhibitory effect of METTL3 knockdown on osteogenic differentiation. We found that silencing METTL3 observably inhibited the expression of METTL3 and BMP2, and subsequent Lv-BMP2 transduction markedly restored the protein level of BMP2 without affecting the expression of METTL3 (Figure 6A, 6B). Moreover, the mRNA and protein levels of RUNX2, COL1A1, OPN and OCN were reduced on day 14 after METTL3 knockdown, which was reversed by BMP2 overexpression (Figure 6C, 6D). Furthermore, BMP2 overexpression effectively abolished METTL3 silence-induced decrease of osteogenic phenotype and matrix mineralization by facilitating ALP activity and staining and ARS accumulation (Figure 6E–6H).

### PiR-36741 affected the osteogenic differentiation of BMSCs by regulating BMP2 expression

**Figure 7:**
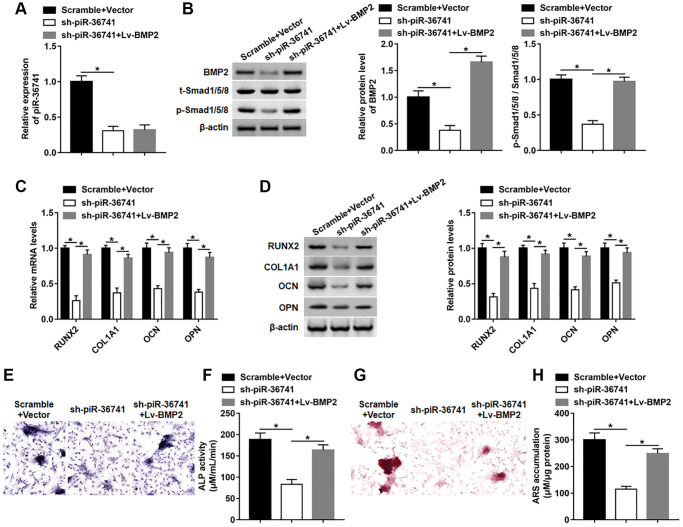
**piR-36741 affected the osteogenic differentiation of BMSCs by regulating BMP2 expression.** BMSCs were infected with Lv-sh-piR-36741 alone or together with Lv-BMP2, and then cultured in osteogenic differentiation medium for 14 days. (**A**, **B**) The expression of piR-36741 and the protein levels of BMP2, p-Smad1/5/8 and Smad1/5/8 were measured on day 14. (**C**, **D**) The expression of METTL3, RUNX2, COL1A1, OCN and OPN were detected on day 14. (**E**) Images of ALP staining (100×). (**F**) ALP activity was determined on day 14. (**G**) Images of ARS staining (100×). (**H**) Quantitative analysis of ARS accumulation on day 14. *N* = 5 in each group. ^*^P < 0.05. Each test was independently repeated at least three times.

To investigate whether piR-36741 regulated osteogenic differentiation by affecting BMP2 expression, BMSCs were treated with piR-36741 shRNA alone or together with BMP2 overexpression vector. Interference with piR-36741 dramatically suppressed the expression of piR-36741 and BMP2 protein, while the subsequent Lv-BMP2 transduction only up-regulated the level of BMP2 protein without altering piR-36741 expression (Figure 7A and 7B). And the high expression of BMP2 restrained the downregulation of phosphorylated-Smad1/5/8 (p-Smad1/5/8) induced by silencing piR-36741 (Figure 7B). Moreover, the mRNA and protein levels of RUNX2, COL1A1, OPN and OCN were decreased by knockdown of piR-36741, which was abolished by overexpressing BMP2 (Figure 7C and 7D). In addition, overexpression of BMP2 reversed the reduction in osteogenic phenotype and matrix mineralization induced by silencing piR-36741 by increasing ALP activity and staining and ARS accumulation (Figure 7E–7H).

### Overexpression of piR-36741 alleviated ovariectomy-induced osteoporosis in mice

**Figure 8:**
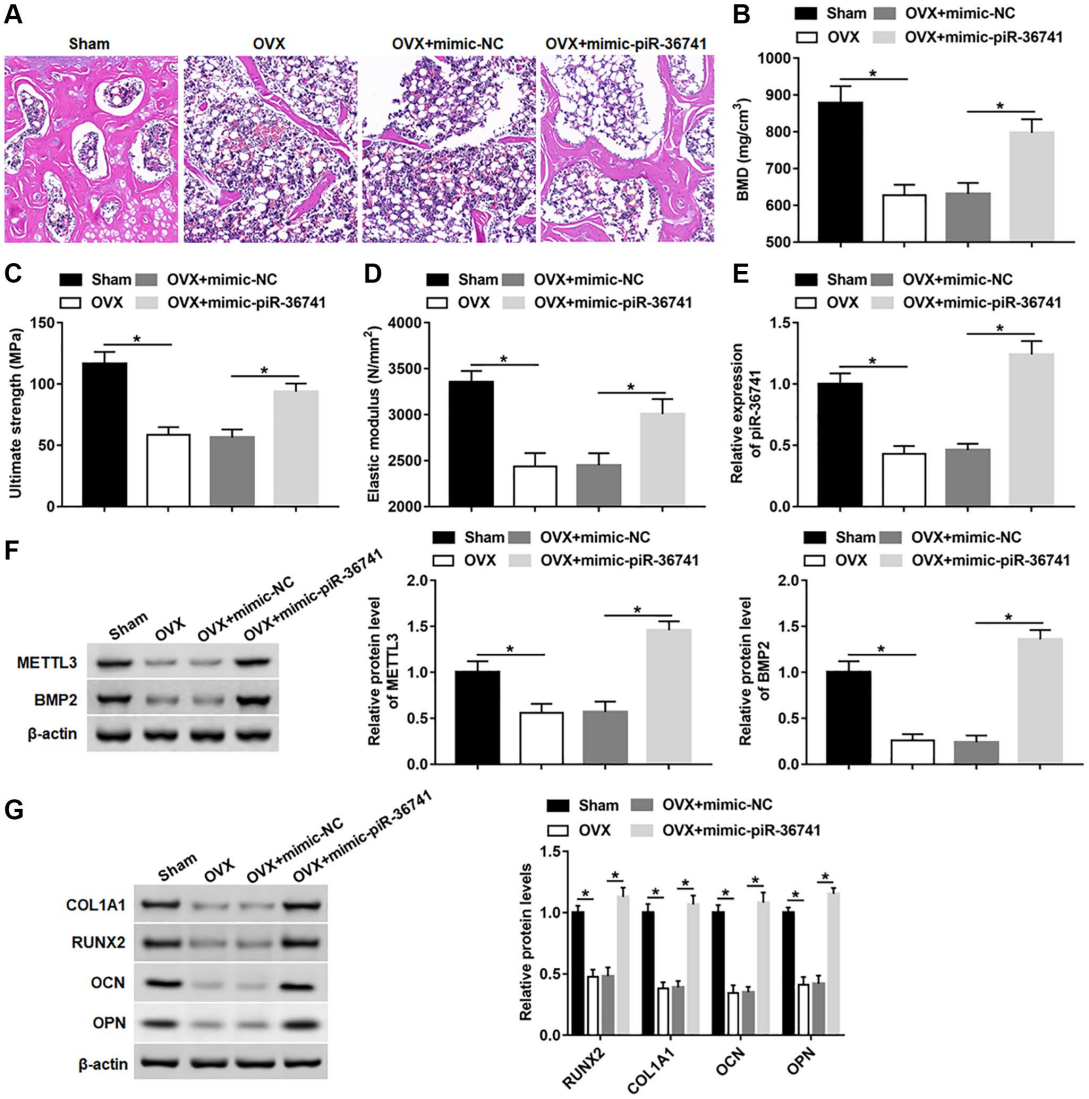
**Overexpression of piR-36741 alleviated ovariectomy-induced osteoporosis in mice.** Ovariectomy was used to construct a mouse model of osteoporosis. 10 mg/kg mimic-NC or mimic-piR-36741 in 50 μL volumes were respectively injected into mice 14 days after the ovariectomy through the tail vein once a week until the 8th week. (**A**) Representative images of HE staining of mouse distal femur tissue sections (100×). (**B**–**D**) Bone mineral density, bone strength and elastic modulus were evaluated. (**E**, **F**) The expression of piR-36741 and the protein levels of METTL3 and BMP2 in femoral tissues were measured. (**G**) The protein levels of RUNX2, COL1A1, OCN and OPN were analyzed with Western blotting. *N* = 6 in sham group, and *N* = 8 in OVX, OVX+mimic-NC, and OVX+mimic-piR-36741 groups. ^*^P < 0.05. Each test was independently repeated at least three times.

To explore whether piR-36741 could alleviate ovariectomy-induced osteoporosis, we treated OVX mice with mimic-NC and mimic-piR-36741. Compared with the sham group, the skeletal structure of the mice in the OVX group was disorderly and the trabecula was obviously lost (Figure 8A). After the administration of mimic-piR-36741, the bone structure of OVX mice was significantly improved, and the thickness of bone trabecula was increased significantly (Figure 8A). Moreover, overexpression of piR-36741 observably increased the bone density, bone strength and bone elasticity coefficient in osteoporotic mice (Figure 8B–8D). In addition, compared with the sham group, the expression of piR-36741, METTL3, BMP2, RUNX2, COL1A1, OPN and OCN in bone marrow tissues of OVX group was notably down-regulated, which was reversed by administration with mimic-piR-36741 (Figure 8E–8G).

## DISCUSSION

The PIWI-piRNA pathway is considered to be essential for silencing transposable elements through DNA methylation to maintain the genomic integrity of germline stem cells [16]. Recently, emerging evidence has shown that in addition to mammalian germ lines, piRNAs are also exist in normal body cells (such as heart, brain, bone marrow, and other tissues) or cancer cells other than germ cells, and are widely involved in cancer development. For instance, the expression of piRNA-823 is up-regulated in patients with multiple myeloma and is positively correlated with clinical stage [17]. Overexpression of piRNA-8041 can reduce the proliferation of glioblastoma cells, induce cell cycle arrest and apoptosis, and can also significantly decrease the volume of intracranial xenograft tumors in mice [18]. According to a recent report, piR-36741 is significantly related to the TNM staging of clear cell renal cell carcinoma [19]. In addition, the expression of piR-36741 was prominently up-regulated during the osteogenic differentiation of human bone marrow mesenchymal stromal cells [10]. Our results displayed that piR-36741 was up-regulated during the osteogenic differentiation of BMSCs, which was consistent with the previous report. Besides, silencing piR-36741 significantly suppressed the osteogenic differentiation, manifested as reduced osteoblast marker expression, osteogenic phenotype and matrix mineralization. In addition, administration with piR-36741 mimic alleviated ovariectomy-induced osteoporosis in mice.

M6A modification of METTL3-mediated eukaryotic RNA has a broad impact on stable homeostasis, and imbalance of m6A levels may lead to dysfunction or disease [20]. METTL3 is reported to be involved in the regulation of bone biology and osteoporosis. Silencing METTL3 suppressed the osteogenic differentiation of BMSCs, leading to impaired bone formation and bone marrow fat accumulation, and METTL3 overexpression alleviated estrogen deficiency-caused osteoporosis [14]. METTL3 induces m6A methylation of pre-miR-320, and m6A reader protein YTHDF2 binds to methylated pre-miR-320 to induce degradation of pre-miR-320, thereby promoting osteogenic differentiation of BMSCs [21]. Besides, the knockdown of METTL3 stables Smad7 and Smurf1 mRNAs via YTHDF2 involvement, hindering osteoblastic differentiation and Smad-dependent signal transduction [22]. METTL3 expression is upregulated in BMSCs receiving osteogenic induction, and RNA sequencing analysis revealed that a large number of genes affected by silencing METTL3 were related to osteogenic differentiation and bone mineralization [23]. We found that METTL3 expression was increased during osteogenic differentiation of BMSCs, and the knockdown of METTL3 restrained osteoblast marker expression and reduced the osteogenic phenotype.

Recently, the regulatory role of piRNAs on m6A methylation has been explored. piR-30473 is reported to reduce WTAP mRNA attenuation and enhance mRNA stability by binding to the 3′UTR of WTAP to mediate m6A modification in diffuse large B-cell lymphoma cells [24]. A cardiac-hypertrophy-associated piRNA is demonstrated to inhibit the m6A modification of the target gene of METTL3 by competitively binding to METTL3 [15]. In addition, ALKBH5 is reported to promote the osteogenesis of the ligamentum flavum cells by down-regulating BMP2 m6A level and activating the AKT signal pathway [25], suggesting that BMP2 can undergo m6A methylation modification. The results in this study demonstrated that piR-36741 formed a complex with PIWIL4 and competitively bound to METTL3 with BMP2 mRNA, thereby reducing the m6A activity of METTL3 without changing its expression. The decrease of the m6A activity of METTL3 led to a reduction in the m6A level of BMP2 mRNA and declined the degradation of BMP2 mRNA mediated by YTHDF2, thus up-regulating the mRNA and protein expression of BMP2.

The differentiation of osteoblasts is highly regulated by hormones, cytokines and a variety of transcription factors [26]. It is determined that many growth factors play a vital role in regulating the osteogenic differentiation of BMSCs, such as bone morphogenetic protein (BMP) family, Runx2, ALP [27]. BMP2 acts as a positive regulator of osteoblastic differentiation and promotes osteoblastic differentiation by activating the Smads signaling cascade [28, 29]. RUNX2 expression is induced by BMP2 during osteogenic differentiation, and it can regulate the expression of osteogenic markers, such as osteocalcin (OCN), COL1A1, and osteopontin (OPN) [30, 31]. Moreover, the high expression of BMP2 mitigates glucocorticoid-induced osteoporosis [32]. Our data indicated that overexpression of BMP2 reversed the down-regulation of osteoblast markers (including COL1A1, RUNX2, OCN, and OPN) and the reduction of osteogenic phenotype induced by silencing piR-36741/METTL3. Furthermore, the knockdown of piR-36741 notably suppressed the Smad1/5/8 pathway activity, which was abolished by the high expression of BMP2.

In conclusion, our results indicated that piR-36741 had a protective effect on the osteogenic differentiation of BMSCs, and its overexpression could reduce bone loss in mice with osteoporosis. PiR-36741 played its role mainly by impeding METL3-mediated m6A methylation of BMP2 mRNA to up-regulate BMP2 expression. These data may provide a novel molecular target for the treatment of postmenopausal osteoporosis.

## MATERIALS AND METHODS

### BMSC isolation, culture and osteogenic differentiation induction

The bone marrow tissues were obtained from patients with femoral neck fracture and/or femoral head fractures undergoing hip joint replacement, from the Shaanxi Provincial People’s Hospital. A written form of informed consent was obtained from all patients, and the study was approved by the Ethics Committee of Shaanxi Provincial People’s Hospital.

The human bone marrow mesenchymal stem cells (BMSCs) were isolated as previously described [3]. The cells were cultured in α-MEM medium (HyClone, USA) with 10% FBS (Gibco, USA) and 1% penicillin-streptomycin (HyClone, USA) at 37°C in the presence of 5% CO2. To induce osteogenic differentiation, 10 mM β-glycerophosphate (Sigma-Aldrich, USA), 100 nM dexamethasone (Sigma-Aldrich, USA), and 200 μM ascorbic acid (Sigma-Aldrich, USA) were added to the medium. The induction lasted for 14 days, and the medium was changed every 3 days.

### Cell transfection

The piR-36741 mimic (mimic-piR-36741), mimic negative control (mimic-NC), piR-36741 antagomir (antagomir-piR-36741), antagomir negative control (antagomir-NC), YTHDF2 siRNA, and NC siRNA were obtained from GenePharma. 2′-O-methylation modification was performed in all the bases, and cholesterol was added to the 3′ end. pcDNA3.1-mediated METTL3, YTHDF2 and PIWIL4 overexpression vectors (pcDNA-METTL3, pcDNA-YTHDF2, and pcDNA-PIWIL4) were constructed as previously described [33]. Lentivirus-mediated BMP2 overexpression vector (Lv-BMP2; 2 × 10^11^ pfu), and piR-36741, METTL3 and YTHDF2 shRNA, and non-target shRNA lentiviral particles (sh-piR-36741, sh-METTL3, sh-YTHDF2, and scramble; 2 × 10^11^ pfu) were purchased from Invitrogen. The sequences were as follows: mimic-piR-36741–5′- GTT TAG ACG GGC TCA CAT CAC CCC ATA AAC A-3′; mimic-NC-5′-UCC UCC GAA CGU GUC ACG UTT-3′; antagomir-piR-36741–5′-UGU UGG GGU GAU GUG AGC CCG UCU AAA C-3′; antagomir-NC-5′-UUG UAC UAC ACA AAA GUA CUG-3′; sh-METTL3–5′-GCG TGA GAA TTG GCT ATA TCC-3′; scramble-5′- CCG GCA ACA AGA TGA AGA GCA CCA ACT CGA GTT GGT GCT CTT CAT CTT GTT GTT TTT G-3′. Cell transfection was performed using Lipofectamine^®^ 3000 reagent (Thermo, Waltham, MA, USA) according to the manufacturer’s instructions. As shown in Supplementary Figure 1, cells were infected with lv-sh-piR-36741, lv-sh-METTL3, or lv-BMP2 with a multiplicity of infection (MOI) of 50, 100 and 200, and the optimal MOI was 100.

### Reverse Transcription-quantitative PCR (RT-qPCR)

Total RNA was isolated from BMSCs and mouse distal femur tissues using TRIzol reagent (Invitrogen, Carlsbad, CA, USA) in accordance with the manufacturer’s instructions. SuperScript III Reverse Transcriptase (Invitrogen, Grand Island, NY, USA) was used to reversely transcribe RNA into cDNA in accordance with the manufacturer’s instructions. qPCR was carried out by using a One Step SYBR^®^ PrimeScript^™^ PLUS RT-PCR Kit (Takara, Dalian, China). mRNA expression levels were calculated using the 2^−ΔΔCt^ method and normalized versus GAPDH. The reaction conditions were set as follows: incubation at 95°C for 5 min, followed by 35 cycles of 95°C for 10 s and 60°C for 1 min. The primer sequences were as follows: GAPDH: Forward-5′-GGG CAC GAA GGC TCA TCA TT-3′, Reverse-5′-AGA AGG CTG GGG CTC ATT TG-3′; BMP2: Forward-5′- ACT CGA AAT TCC CCG TGA CC-3′, Reverse-5′-CCA CTT CCA CCA CGA ATC CA-3′; RUNX2: Forward-5′-CGA ATA ACA GCA CGC TAT TAA-3′, Reverse-5′-GTC GCC AAA CAG ATT CAT CCA-3′; OCN: Forward-5′-GGC GCT ACC TGT ATC AAT GG-3′, Reverse-5′-GTG GTC AGC CAA CTC GTC A-3′; OPN: Forward-5′- GGA GTT GAA TGG TGC ATA CAA GG-3′, Reverse-5′-CCA CGG CTG TCC CAA TCA G-3′; COL1A1: Forward-5′-GGG TCT AGA CAT GTT CAG CTT TGT G-3′, Reverse-5′-ACC CTT AGG CCA TTG TGT ATG C-3′; U6: Forward-5′- CTC GCT TCG GCA GCA CA-3′, Reverse-5′-AAC GCT TCA CGA ATT TGC GT-3′; piR-36741: 5′- GTTTAGACGGGCTCAC ATCAC-3′.

### Western blotting

Total cell and mouse distal femur tissue extracts were harvested in RIPA buffer, and centrifuged at 4°C for 10 min at 13,000 rpm. Enhanced BCA Protein Assay Kit was used to quantify proteins. Next, 30 μg proteins were separated by 10% SDS-PAGE for 2 h, and then transferred onto PVDF membranes for 1.5–3 h. Then, the membranes were blocked in 5% skim milk for 2 h, probed using primary antibodies at 4°C overnight, and incubated with appropriate HRP-conjugated secondary antibodies at room temperature for 1 h. Signals were measured by using an enhanced chemiluminescence (ECL) reagent (Advansta, Menlo Park, CA) according to the manufacturer’s protocol. Densitometric analysis was carried out by using the Lumino-Image analyzer LAS-3000 system (Fuji Film, Tokyo, Japan). β-catin was used to confirm equal protein loading. Antibodies used in this study were as follows: anti-Collagen I (ab34710; 1:1000; Abcam, Cambridge, UK), anti-RUNX2 (ab76956; 1:1000; Abcam), anti-Osteocalcin (ab93876; 1:1000; Abcam), anti-Osteopontin (ab166709; 1:1000; Abcam), anti-BMP2 (ab14933; 1:500; Abcam), anti-METTL3 (ab240595; 1:5000; Abcam), anti-METTL14 (ab223090; 1:3000; Abcam), anti-WTAP (ab155924; 1:2000; Abcam), anti-ALKBH5 (ab69325; 1:1000; Abcam), anti-FTO (ab124892; 1:5000; Abcam), anti-PIWIL1 (ab181056; 1:5000; Abcam), anti-PIWIL2 (ab181340; 1:1000; Abcam), anti-PIWIL3 (ab77088; 1:500; Abcam ), anti-PIWIL4 (ab111714; 1:1000; Abcam), β-actin (#12262; 1:1000; Cell Signaling Technology, MA, USA).

### Alkaline phosphatase (ALP) staining

After 10 days of induction of BMSCs, the culture medium was discarded, and the cells were washed with deionized water and fixed at room temperature with 10% paraformaldehyde for 15 min. Then, cells were stained at 37°C with a solution of 5-bromo-4-chloro-3-indolyl-phosphate/nitro-blue tetrazolium solution (Sigma-Aldrich Co., St Louis, MO, USA) for 30 min and imaged under a microscope (Eclipse TS100; Nikon, Melville, NY, USA).

### ALP activity

The BMSCs washed with PBS were lysed with 1% Triton X-100 for 15 min, and then centrifuged at 10,000 rpm/min for 5 min. The supernatant was collected and analyzed for ALP activity with Alkaline phosphatase ELISA Kit (Beyotime Biotechnology). The absorbance value at 405 nm was measured with a microplate reader, and the ALP activity was calculated based on the absorbance.

### Alizarin red staining (ARS)

BMSCs were fixed with 4% formaldehyde at room temperature for 30 min and washed 3 times with PBS. Next, the cells were treated at room temperature with 40 mM ARS solution (S0141; Cyagen) for 5 min, and the stained cells were observed under a light microscope (Eclipse TS100). The alizarin red-positive area indicating osteogenesis was calculated using Image J software.

### RNA immunoprecipitation assay (RIP)

According to the manufacturer’s protocol, the RIP analysis was performed using Magna RIP RNA-binding Protein Immunoprecipitation Kit (Millipore, Bedford, MA, USA). Cells were collected and lysed using RIP lysis buffer. The lysate was then incubated overnight with anti-Ago2 or IgG antibodies at 4°C. The immunoprecipitated RNA was eluted and analyzed with RT-qPCR.

### MeRIP-qPCR

Total RNA was extracted from BMSCs and fragmented. A part of the RNA sample was used as input, and the remaining sample was incubated with beads conjugated with m6A-antibody in immunoprecipitation buffer at 4°C overnight. The m6A-containing RNA samples were then eluted from the beads. Gene-specific primers were used for RT-qPCR of the input control and m6A immunoprecipitated samples.

### M6A quantification

An EpiQuik m6A RNA Methylation Quantification Kit (Colorimetric) (Epigentek, Germany) was used to analyze the global m6A levels of mRNA in BMSCs according to the manufacturer’s protocol. Sample was analyzed by using Poly (A) RNA-(200 ng).

### Biotin-labeled piRNA pull-down assay

The 3′-end biotinylated piR-36741 and its NC were obtained from GenePharma. The cells transfected with biotinylated piR-36741 or its NC were collected and washed with PBS. The cells were then resuspended in a lysis buffer and incubated on ice for 10 min. After centrifugation, 50 μl of the lysate was taken as input, and the remaining lysate was incubated with streptavidin-sepharose beads (Sigma). After the pellet was washed 3 times, it was boiled in SDS-PAGE loading buffer. Finally, the precipitated protein was analyzed by Western blot.

### Co-immunoprecipitation (Co-IP)

Cells were collected and resuspend in IP lysis buffer, and 1 μg of anti-METTL3 (ab240595; Abcam) antibody or 1 μg of anti-PIWIL4 (ab180867; Abcam) antibody were added into the lysate and incubated overnight at 4°C. Pretreated Protein A/G Plus -Agarose Beads (SC-2003, Santa Cruz) were added to the mixture at 4°C and gently shaken for 2–4 hours. The beads were washed for 4 times with corresponding IP buffer, and then suspended in SDS-PAGE loading buffer and boiled for 10 min. The homotypic IgG served as a negative control.

### Histological analysis

The femurs of the test mice were separated and the femoral tissues were immediately fixed in 4% paraformaldehyde. After a series of ethanol dehydration, the sample was embedded in paraffin. 4 μm-thick tissue sections were stained with haematoxylin and eosin (H&E) at 30°C for 120 min, and bone quality and density were analyzed with a microscope (Eclipse TS100).

### The measurement of bone mineral density (BMD), bone strength and elastic modulus

The bone density of femur was determined by using the Mindways QCT bone density measurement system. The ElectroForce Dynamic Mechanics Test System (Bose ElectroForce^®^ 3230) was used to measure the bone strength and elastic modulus of the femur.

### Mouse model of osteoporosis

Experiments were performed following the Guidelines of the Institutional Animal Use and Care Committee of Shaanxi Provincial People’s Hospital. Female C57BL/6 mice were fed under pathogen-free conditions and kept in a controlled environment (temperature 23 ± 1°C; humidity 50–60%), and subjected to a 12-hour light/dark cycle artificial simulation. Eight-week-old mice were selected for surgery and randomly divided into sham group (*n* = 6) and ovariectomized model group (OVX, *n* = 24). Mice in the OVX group were anesthetized with 5% ketamine and bilateral ovaries were removed after tubal ligation as previously reported [2]. After complete hemostasis, the wound was sutured. Mice in the sham group had only adipose tissue removed near their bilateral ovaries. The mice were raised for another 8 weeks and then sacrificed.

On the 14th day after surgery, 16 mice from the OVX group were randomly selected and divided into 2 groups with 8 mice in each group. 10 mg/kg mimic-NC or mimic-piR-36741 were respectively injected into the two groups of mice through the tail vein once a week until the 8th week.

### Statistical analysis

All data were expressed as mean ± SEM and analyzed with SPSS 22.0. The Shapiro-Wilk test was performed to evaluate whether the data was normally distributed. Levene’s test was performed to analyze the homogeneity of variances. Statistical analysis was carried out using Student’s *t*-test between two groups and one-way analysis of variance (ANOVA) among multiple groups in accordance with data normal distribution and homogeneity of variances. Statistical significance was set at *P* < 0.05.

### Availability of data and materials

All data used during the current study are available from the corresponding author on reasonable request.

### Ethics approval and consent to participate

This research was approved by the Ethics Committee of Shaanxi Provincial People’s Hospital (SRMYY-2019-043). All patients obtained informed consent before surgery. All animal experiments were in accordance with the guide for the care and use of laboratory animals established by United States National Institutes of Health (Bethesda, MD, USA).

## Supplementary Materials

Supplementary Figure 1
