# Supplementary material for: piRNA-36741 regulates BMP2-mediated osteoblast differentiation via METTL3 controlled m6A modification
Source: Aging (Albany NY). 2021 Oct 13;13(19):23361–75. doi: 10.18632/aging.203630 (PMC8544320; doi:10.18632/aging.203630)
Supplement: Supplementary Figure 1 [file aging-13-203630-s001.pdf]

SUPPLEMENTARY FIGURE

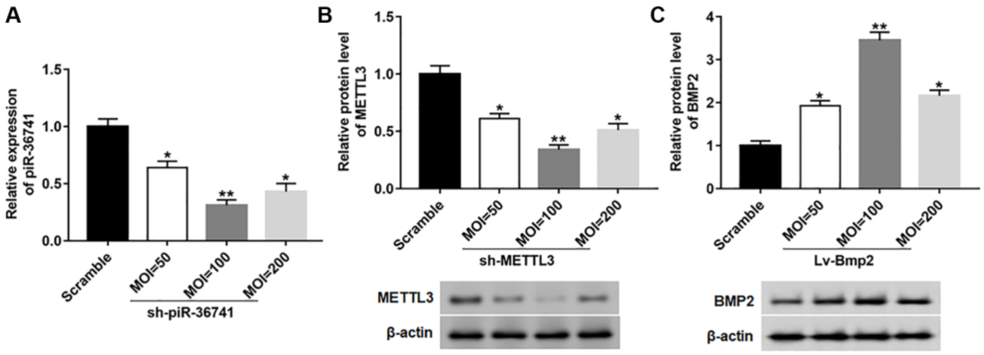

Supplementary Figure 1. Optimizing the multiplicity of infection (MOI) for lentivirus. (**A**) BMSCs were infected with Lv-sh-piR-36741 with a MOI of 50, 100, or 200, and the expression of piR-36741 was measured with RT-qPCR. (**B**, **C**) BMSCs were infected with Lv-sh-METTL3/Lv-BMP2 with a MOI of 50, 100, or 200, and the protein levels of METTL3/BMP2 were determined with Western blotting. *N* = 5 in each group. $^{*}P < 0.05$, $^{**}P < 0.01$. Each test was independently repeated at least three times.
